# Association of GAS6, AXL, and GAS6-AS lncRNAs with nephropathy in Egyptian patients with type 2 diabetes mellitus: a case–control observational study

**DOI:** 10.1038/s41387-025-00400-y

**Published:** 2025-11-13

**Authors:** Tarek Kamal Motawi, Dina Sabry, Nancy Mamdouh Ahmed, Nancy Nabil Shahin

**Affiliations:** 1 Department of Biochemistry, Faculty of Pharmacy, Cairo University, Cairo, 11562 Egypt; 2 Department of Medical Biochemistry and Molecular Biology, Faculty of Medicine, Cairo University, Cairo, 11562 Egypt; 3 Department of Medical Biochemistry and Molecular Biology, Faculty of Medicine, Badr University in Cairo, Cairo, 11829 Egypt; 4 Department of Biochemistry, Faculty of Pharmacy, Nahda University, Beni Suef, 62761 Egypt

**Keywords:** Diabetes complications, Genetics

## Abstract

**Background/objectives:**

Diabetic nephropathy (DN) is a prevalent microvascular diabetic complication that is not totally unveiled. In this study, we considered GAS6, AXL, GAS6-AS1, and GAS6-DT as possible early diagnostic biomarkers of DN.

**Subjects/methods:**

The study included 70 patients with normoalbuminuria type 2 diabetes (DM), 70 patients with microalbuminuria type 2 diabetes (DN), and 60 apparently healthy controls. Fasting plasma glucose, glycosylated hemoglobin, and plasma creatinine were enzymatically assayed. Albuminuria, plasma GAS6, and AXL levels were determined using ELISA. Long non-coding RNAs GAS6-AS1 and GAS6-DT levels were determined in blood using qRT-PCR. Several bioinformatics databases were employed to suggest interactions with the studied biomolecules.

**Results:**

GAS6 and AXL were downregulated in DM + DN compared to controls, being the lowest in DN (*p* < 0.0001). GAS6-DT was upregulated in DM + DN compared to controls, being the highest in DN (*p* < 0.0001). GAS6-AS1 was higher in DN than in controls (*p* = 0.013). GAS6, AXL, and GAS6-DT showed fair-to-moderate significant correlations with HbA1c, fasting glucose, creatinine, and albuminuria. ROC curves showed remarkable diagnostic power of GAS6, AXL, and GAS6-DT (AUC = 0.72–1.0), but not GAS6-AS1, in DN and DM, with moderate-to-excellent agreement with conventional diagnostics.

**Conclusions:**

The current findings emphasize the significance of the GAS6/AXL pathway in DM and DN progression, where GAS6, AXL, and GAS6-DT showed significantly altered values in DM, and further in DN, with notable diagnostic power for both diseases. To date, this is the first study confirming the diagnostic power of AXL and GAS6-DT in DN and DM. Future studies are warranted to evaluate therapeutically targeting this pathway to manage DN.

## Introduction

Globally, type 2 diabetes prevalence is high, and rising even more in all world regions. Egypt is ranked tenth among the top ten world countries, regarding the number of adults with diabetes, and is ranked second in the Middle East and North Africa region [1].

Diabetic nephropathy (DN) is a widespread microvascular diabetic complication. So far, its etiology and pathogenesis are still indeterminate. It has been related to inflammatory responses, oxidative stress, and hemodynamic disorders resulting from hyperglycemia. Renal tissue changes occur, including glomerular basement membrane thickening, mesangial expansion, glomerulosclerosis, renal vessel and tubulointerstitial lesions, as well as increased fibrosis in renal cells, and kidney function decline. Many patients with diabetic kidney disease are susceptible to rapid progression into end-stage renal disease within months [2].

Several strategies are pursued to identify novel DN biomarkers in blood and urine [3] for the early detection of DN stages and kidney function decline. These include various omics approaches such as metabolomics, peptidomics, multi-omics integration [4], and the study of extracellular vesicles [5], as well as targeted investigations of candidate biomarkers [6]. However, microalbuminuria is still regarded as the “gold standard” for diagnosis and estimation of DN prognosis [7].

Growth arrest-specific gene 6 (GAS6), a growth-factor-like plasma protein, interacts with the TAM family of receptor tyrosine kinases (Tyro3, Mer, and preferentially binds to AXL). The GAS6/TAM signaling controls processes like cell migration, proliferation, adhesion, and survival. Activation of AXL induces phagocytosis and decreases the expression of proinflammatory cytokines. TAM receptor extracellular domains are cleaved proteolytically by metalloproteases forming soluble forms of the receptors, e.g., sAXL, which circulate in plasma and function as decoys to inactivate their circulating ligand [8].

GAS6 and AXL are expressed in the kidneys. Plasma GAS6 level was significantly lower in patients with diabetes than in healthy controls and decreased further in patients with diabetes and albuminuria than in patients with diabetes and normoalbuminuria [8]. Other studies mentioned that plasma GAS6 level was elevated significantly in participants with diabetes and microalbuminuria than in those with diabetes and normoalbuminuria [9]. GAS6/AXL signaling induced mesangial proliferation and glomerular hypertrophy in DN through the activation of the AKT/mTOR pathway, promoting glomerular injury [10]. Thus, GAS6 was suggested as a potential noninvasive diagnostic biomarker for early diagnosis of DN [8].

Long non-coding RNAs (lncRNAs) are transcripts of 200 nucleotides to 100 kilobases, which do not encode proteins. They assume distinct three-dimensional structures, which allow them to interact with DNA, mRNA, proteins, and other non-coding RNAs. LncRNAs coordinate gene expression, from nuclear and cytoplasmic epigenetic processes (miRNA sponging and chromatin remodeling) to mRNA translation, splicing, and decay. They are involved in cellular processes, including differentiation, proliferation, and apoptosis. Their dysregulation can contribute to a multitude of human diseases, including kidney disease pathogenesis and progression [2] and diabetic complications [11]. The high stability of lncRNAs in biofluids and their ease of detection make them useful predictive biomarkers. LncRNAs may serve as biomarkers as well as therapeutic targets for complications of diabetes [11]. LncRNAs are suggested as targets to develop next-generation RNA-based therapy for kidney diseases [2]. Several lncRNAs were upregulated in DN, such as plasmacytoma variant translocation 1 (PVT1), stress-regulated lncRNA hosting a microRNA megacluster (Lnc-MGC), noncoding RNA within the intron region of Erbb4 (Erbb4-IR), metastasis-associated lung adenocarcinoma transcript 1 (MALAT1), and antisense noncoding RNA in the INK4 locus (ANRIL), while taurine upregulated gene 1 (Tug1) and long intergenic non-protein coding RNA 1619 (Linc01619) were downregulated [12].

GAS6-AS1 is a lncRNA with 902 base pairs and 5 exons, located at 13q34, and is transcribed antisense to GAS6 [7]. GAS6-AS1 level was inversely correlated with GAS6 mRNA level [13], and it also affects AXL level [14]. GAS6-DT, previously named GAS-AS2, is a lncRNA with 1942 base pairs and 2 exons. It upregulates both GAS6 and its TAM receptors to promote cellular survival [15]. Unlike well-characterized lncRNAs like MALAT1, NEAT1, and PVT1, which are broadly associated with various pathological conditions [16, 17], GAS6-DT and GAS6-AS1 are relatively novel lncRNAs that are specifically linked to the regulation of the GAS6 gene, suggesting that they may have more targeted and distinct regulatory roles in disease states. Both lncRNAs have not yet been considered in diabetes or DN studies.

The aim of the current work was to investigate the potential role of GAS6, AXL, GAS6-AS1, and GAS6-DT in type 2 diabetes development and progression to DN, as well as their potential utility as biomarkers for DN in Egyptian patients.

## Subjects and methods

### Human samples

A total of 140 patients were enrolled from the Internal Medicine Department, Faculty of Medicine, Cairo University during the period from June 2019 to March 2020. Sixty sex- and age-matched apparently healthy volunteers were chosen as controls (Supplementary Fig. 1). Criteria for exclusion were pregnancy, lactation, active urinary tract infection, type 1 diabetes, diabetic ketoacidosis, autoimmune diseases, malignant tumors, history of myocardial infarction, psychiatric diseases, thyroid disorders, use of anticoagulant therapy, or vitamin K supplementation.

An informed written voluntary consent was signed by all participants before enrollment in the study, and their privacy rights have been observed. Our research protocol was approved in May 2019 by the Research Ethics Committee for experimental and clinical studies, Faculty of Pharmacy, Cairo University, Egypt under approval number BC (2438). The study conformed to International and Egyptian ethical and regulatory standards for research involving humans, issued by the Council for International Organizations of Medical Sciences, as last updated in 2016. We followed the ethical standards of the 2013 latest amendments of the Helsinki Declaration throughout all investigational protocols.

A 24-h urine sample and a 5 mL fasting venous blood sample were collected in EDTA tubes, 3 mL were centrifuged for 15 min at 2000 × *g* to obtain plasma, and 2 mL remained as whole blood, all stored in aliquots at −80 °C for further analysis. Patients were subclassified based on their glycosylated hemoglobin and albuminuria into 70 patients with type 2 diabetes and normoalbuminuria as the DM group, and 70 patients with diabetes suffering microalbuminuria (30–300 mg/day) as the DN group. All samples were blind while analyzing.

### Biochemical parameters

Albuminuria was determined in a 24-h urine sample by an enzyme-linked immunosorbent assay using a quantitative sandwich technology, with an ELISA kit supplied by Wuhan Fine Biotech Co., Ltd., Hubei, China (catalog no.: EH2613). Plasma creatinine level was determined using a kinetic colorimetric kit provided by Spectrum Diagnostics, Cairo, Egypt (catalog no.: 234 001). For quantification of glycosylated hemoglobin in whole blood, an enzymatic assay kit provided by Crystal Chem, Elk Grove Village, Illinois, USA (catalog no.: 80099) was used. Plasma glucose level was quantified by a liquizyme glucose colorimetric kit provided by Spectrum Diagnostics, Cairo, Egypt (catalog no.: 250 007). All procedures were carried out in compliance with the manufacturer’s protocol.

### qRT-PCR of lncRNAs

Total RNA was extracted from blood samples using Direct-zol RNA Miniprep Plus provided by Zymo Research Corp., Orange, California, USA (catalog no.: R2072). The quantity and quality of the isolated RNA were assessed using a Beckman dual spectrophotometer (USA). SuperScript IV One-Step RT-PCR kit provided by Thermo Fisher Scientific, Waltham, Massachusetts, USA (catalog no.: 12594100), was used for reverse transcription of extracted RNA and PCR in a single reaction tube using gene-specific primers as follows: GAS6-AS1, forward: 5’-GTGGGTACTGCATTCCTACCG-3’, reverse: 5’-CTCTCCTCTGATGGCAGGAC-3’; GAS6-DT, forward: 5’-AAGGAGGACGCAATACC-3’, reverse: 5’-TCTCATCCCAAACCTCCACA-3’; β-actin, forward: 5’-GGCGGCACCACCATGTACCCT-3’, reverse: 5’-AGGGGCCGGACTCGTCATACT-3’. The relative quantification of each target gene was computed and normalized to β-actin housekeeping gene compared to a control sample according to the calculation of ΔΔCt = [(Ct target, Sample) − (Ct ref, Sample)] − [(Ct target, Control) − (Ct ref, Control)]. Fold change = 2^−ΔΔCt^.

### Determination of GAS6 and AXL

Sandwich ELISA kits provided by SunLong Biotech Co., Zhejiang, China were used to quantify the human growth arrest specific protein 6 (GAS6) and the human tyrosine-protein kinase receptor UFO (AXL) levels in plasma samples (catalog no.: SL2945Hu and SL2987Hu, respectively) in compliance with the manufacturer’s instructions.

### In silico bioinformatics analyses

To investigate the possible interactions between our studied parameters and other biomolecules that may be related to the diseases under study, several databases were used. NPInter (http://bigdata.ibp.ac.cn/npinter5/) documents the functional interactions between noncoding RNAs and biomolecules. miRTarBase is a resource for experimentally validated miRNA–target interactions (https://mirtarbase.cuhk.edu.cn/). TargetScan (https://www.targetscan.org/) predicts biological miRNA targets by evaluating the presence of conserved 6mer, 7mer, and 8mer sites that match each miRNA seed region. Only predicted interactions with conserved sites were included for further literature review. PathVisio biological pathway editor version 3.0.0+ was then used to draw a figure summarizing these interactions.

We also used the KEGG PATHWAY Database to demonstrate whether GAS6/AXL were functionally connected to key pathways and molecules implicated in DN.

To ensure the stability and suitability of Gas6 and AXL as biomarkers, the potential impact of non-synonymous SNPs (nsSNPs) on their stability and function was assessed using bioinformatics and computational tools, following methodologies applied in a previous study [18]. Multiple tools were employed for each analysis to enhance the precision and reliability of the results. The databases used were Entrez and UniProt to obtain nucleotide and amino acid sequences, dbSNP and Ensembl VEP for SNP amino acid position and change, SIFT and PROVEAN as sequence homology-based tools, PolyPhen and PredictSNP for Structure homology-based prediction, PANTHER and Phobius to check the conservation of amino acids, and finally I-mutant and MUpro to predict if protein stability was affected by amino acid substitution.

### Statistical analysis

Statistical analyses were performed using SPSS (Chicago, IL, USA) software version 26 for Microsoft Windows. The normality of data was verified by the Kolmogorov–Smirnov test. Data were expressed as median (25th–75th percentile) for continuous parameters and as absolute (*n*) and relative (%) frequency for the categorical variables. Comparisons of the continuous parameters were performed using Kruskal–Wallis one-way analysis of variance between more than two groups, followed by pairwise post hoc Bonferroni comparisons. For comparing categorical data, the chi-square test was used, and Fisher’s exact test was used when >20% of cells had expected frequencies <5. Spearman correlation coefficients (rho) were used to describe the power and direction of relationships between continuous variables, and the connections were plotted as an undirected network graph, with the width of edges proportional to the correlation strength. Receiver operating characteristic (ROC) analyses were employed to assess possible diagnostic performance of suitable parameters, expressed as area under the curve (AUC), sensitivity, specificity, and cut-off values (as determined by Youden index), where AUC ≥ 0.9 is considered excellent, 0.9 > AUC ≥ 0.8 is considered good, 0.8 > AUC ≥ 0.7 is considered fair, and 0.7 > AUC ≥ 0.5 is considered poor. For inter-rater reliability tests, Cohen’s kappa was carried out to assess the level of agreement between two diagnostic parameters, where Kappa values between 0 and 0.2: slight agreement, 0.21 and 0.4: fair agreement, 0.41 and 0.60: moderate agreement, 0.61 and 0.80: substantial agreement, 0.81 and 1: almost perfect or excellent agreement. A probability level of *p* < 0.05 (two-tailed) was set as statistically significant in all analyses. G∗power software version 3.1.9.7 was used to calculate sample size. Assuming the following criteria: studied groups = 3, population variance SD = 0.5, medium effect size *f* = 0.25, *α* error probability 0.05, and type II error *β* = 0.2, the study required a minimum total sample size of 159 individuals to achieve two-tailed power (1 − *β*) = 0.8. Also, an average total sample size of 200 was expected to achieve 0.9 power at 0.25 effect size for the different types of statistical assays used in the study. The computed post hoc achieved power was exactly 0.89 for the total sample size of 200 participants.

## Results

### Demographic data and biochemical parameters in the studied groups

A total of 140 patients were enrolled in this study, subclassified into 70 patients with type 2 diabetes and normoalbuminuria (DM group) and 70 patients with type 2 diabetes and microalbuminuria (DN group). They were compared to 60 apparently healthy volunteers (Control group). The 200 individuals in all groups were sex- and age-matched. Their demographic criteria and biochemical parameters are summarized in Table 1.

**Table 1:** Demographic criteria and biochemical parameters in the studied groups.

	Control (60)	DM (70)	DN (70)	Test statistic	*p*
Age (years)	40 (32–45)	44 (35–48)	42 (36-47.25)	2.202	0.113
Sex				4.738	0.094
Male	40 (34.2%)	34 (29.1%)	43 (36.8%)		
Female	20 (24.1%)	36 (43.4%)	27 (32.5%)		
BMI (kg/m^2^)	24 (21-26.23)	33 (30–36)^a^	22 (19-23.5)^a,b^	137.860	<0.0001
F_Glucose (mg/dL)	92 (75–97)	164 (138.5 -239)^a^	267.5 (205.75-302.5)^a,b^	138.184	<0.0001
HbA1c (%)	5.1 (4.5–5.4)	7.2 (6.7–8.23)^a^	8.5 (8.4–8.9)^a,b^	145.343	<0.0001
Creatinine (mg/dL)	0.8 (0.7–1.06)	1.0 (0.7–1.2)	3.22 (2.8–4.13)^a,b^	137.690	<0.0001
Albuminuria (mg/day)	4.15 (3.8–4.4)	5.5 (3.11–7.73)	58 (45–75)^a,b^	139.999	<0.0001
GAS6 (pg/mL)	251.6 (222.8–315.6)	211 (184.75–264)^a^	104.7 (84.03–125.33)^a,b^	132.857	<0.0001
AXL (pg/mL)	286.95 (241.7–339.18)	220.95 (166.15–259.35)^a^	94.2 (50.98–137.45)^a,b^	131.363	<0.0001
GAS6-AS1 (fold change)	1.0 (0.5–1.48)	1.3 (0.23–2.15)	1.6 (0.78–2.14)^a^	8.680	0.013
GAS6-DT (fold change)	1.02 (0.26–2.35)	7.52 (4.45–9.03)^a^	16.54 (9.32–35.99)^a,b^	151.317	<0.0001

Data are expressed as median (25th–75th percentile), analyzed by Kruskal–Wallis test, then pairwise post hoc Bonferroni comparisons, denoted as: “a” is significantly different from the control group, and “b” is significantly different from the DM group. Sex is expressed as count and percentage; *n* (%), compared by the chi-square test.

*DM* diabetes mellitus group, *DN* diabetic nephropathy group, *F* fasting, *HbA1c* glycosylated hemoglobin.

Significance was considered at *p* < 0.05 (two-tailed).

### GAS6/AXL pathway-related parameters

GAS6 and AXL levels showed a highly significant decrease in the DN group than in the DM group, which, in turn, showed a significant decrease than in the controls at *p* < 0.0001. LncRNA GAS6-AS1 showed significantly higher values in the DN group in comparison with controls, but the increase was not significant compared to the DM group. LncRNA GAS6-DT showed a highly significant increase in the DN group than in the DM group, which, in turn, displayed significantly higher GAS6-DT levels than the controls at *p* < 0.0001 (Table 1).

### Spearman correlations of continuous parameters

**Fig. 1:**
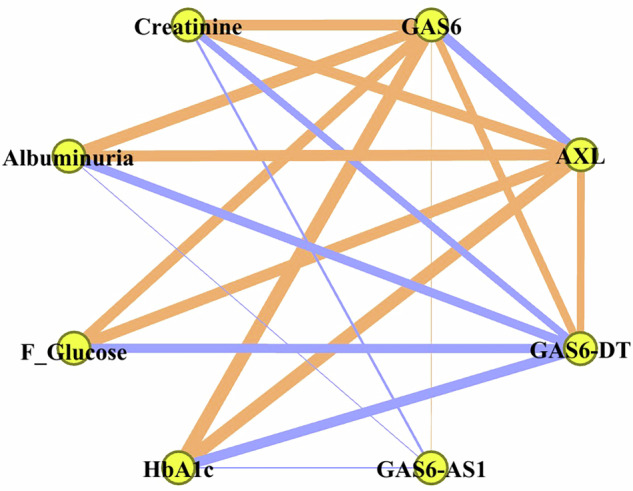
Spearman correlation analysis presented as an undirected network graph. Orange lines represent negative correlations, blue lines represent positive correlations, and the width of the line is proportional to the absolute value of Spearman coefficient, thick lines here represent │0.5│ ≤ rho ≤ │0.8│ while thin lines here represent │0.2│ ≤ rho ≤ │0.3│. Only significant correlations at *p* < 0.05 with absolute coefficient >0.2 were presented.

Applying partial correlations to all 200 cases under study with controlling for age, sex, and BMI, fair-to-moderate significant correlations were found between GAS6, AXL, GAS6-DT, and the continuous parameters (HbA1c, fasting glucose, creatinine, and albuminuria), while GAS6-AS1 showed only weak correlations with others (Supplementary Table 1 and Fig. 1).

### ROC curves of parameters related to the GAS6/AXL pathway

**Fig. 2:**
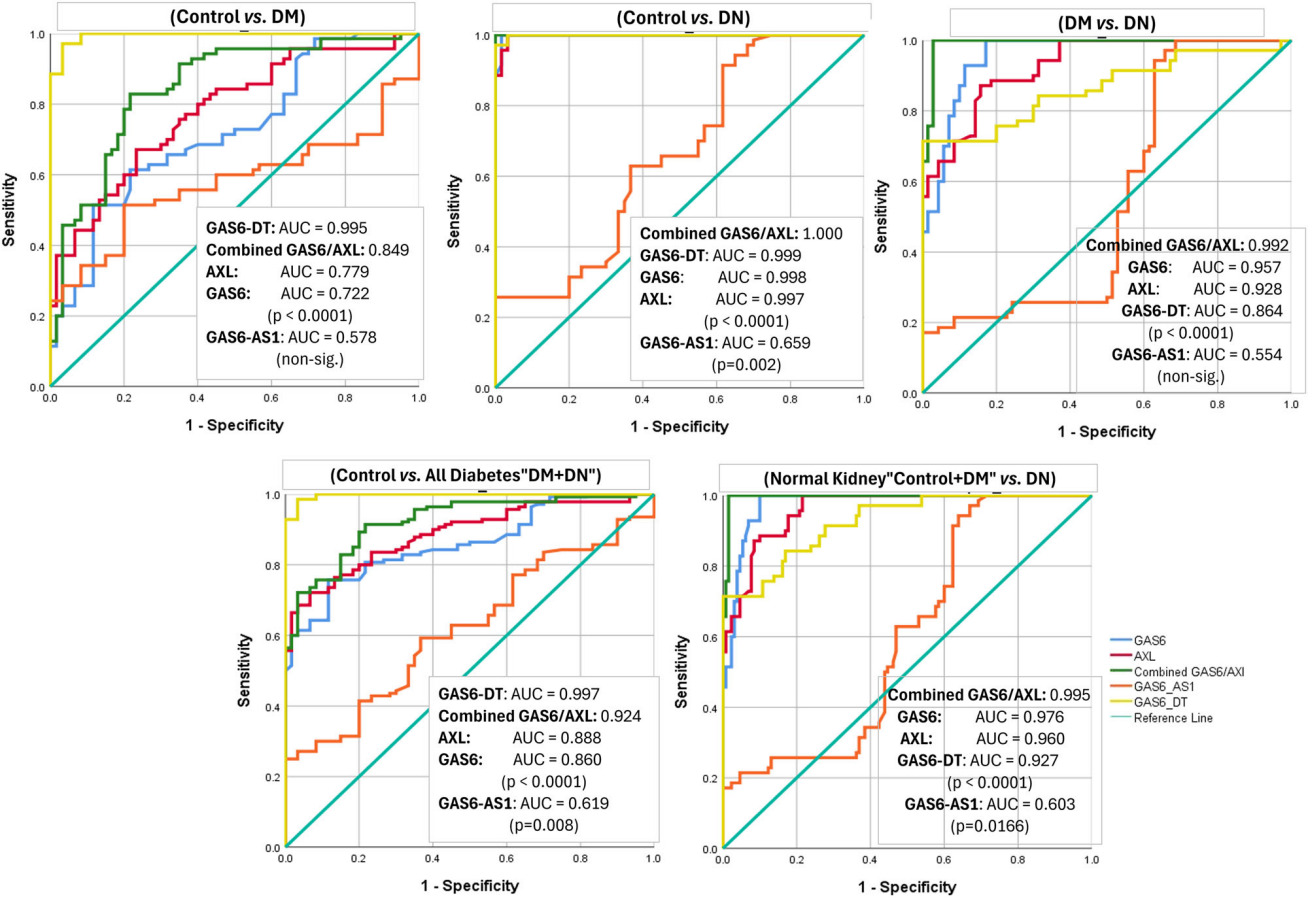
ROC curves of GAS6/AXL-related parameters discriminating diabetes and nephropathy between the different studied groups.

Applying ROC curves (Fig. 2 and Supplementary Table 2), GAS6 showed excellent differentiation of DN patients from others at a cut-off of 174.65 pg/mL, with area under the curve (AUC) exceeding 0.9, specificity ranging from 82.8 to 98.3%, and 100% sensitivity for all 3 possibilities of discriminating DN from healthy control, DM, and from all normal kidney cases (control+DM). It showed fair-to-good discrimination of controls from DM and from pooled patients with diabetes (DM + DN) with AUC of 0.72 and 0.86, respectively, but with lesser sensitivity and specificity. Similar findings were observed with AXL, which could discriminate DN from healthy control, DM, and from normal kidney subjects (control+DM) with an AUC exceeding 0.9, in addition to fair-to-good discrimination of controls from DM and from pooled patients with diabetes (DM + DN) with AUC of 0.78 and 0.89, respectively. Combining GAS6 and AXL by binary logistic regression, followed by ROC curve analysis, the combination enhanced their discriminating power expressed as AUC, sensitivity, and specificity, for all groupings, better than either GAS6 or AXL alone.

GAS6-AS1 had statistically significant poor discriminative power with an AUC < 0.7 to differentiate patients with nephropathy from controls, but it could not discriminate normal kidney DM from controls nor from DN (p > 0.05). For GAS6-DT, it could discriminate patients with diabetes from others at cut-off values greater than 3.29 folds, with AUC > 0.99, sensitivity >97% and 96.67% specificity for all 3 possibilities of discriminating controls from DM, DN, and pooled patients with diabetes (DM + DN). It also showed good to excellent discrimination of DN from DM (patients with diabetes and normal kidney) and from pooled subjects with normal kidney (control + DM) at cut-off values greater than 11.21 folds, with AUC of 0.864 and 0.927, respectively, with 71.43% sensitivity and 100% specificity.

### Agreement level between GAS6-related parameters and conventional parameters as diagnostic tests for diabetes and DN

To investigate the agreement level between the studied diagnostic parameters and the conventional gold standards, cases were re-classified based on the cut-offs of the different parameters, as obtained from ROC analysis, then Cohen’s kappa was calculated. As diagnostic tests for DN, GAS6 had excellent agreement, while AXL and GAS6-DT had substantial agreement with microalbuminuria and creatinine. As diagnostic tests for diabetes mellitus, GAS6-DT had excellent agreement, whereas GAS6 and AXL had moderate agreement with HbA1c and fasting glucose. GAS6-AS1 agreement was very weak in both scenarios (Table 2).

**Table 2:** Agreement level between GAS6-related parameters and conventional parameters [(HbA1c and fasting glucose) as diagnostic tests for diabetes mellitus, and (microalbuminuria and creatinine) as diagnostic tests for diabetic nephropathy].

		Conventional parameters: microalbuminuria or creatinine (1.2 mg/dL)		Kappa			Conventional parameters: HbA1c = 5.7% or F_Glu = 100 mg/dL		Kappa
		Normal kidney (130)	Nephropathy (70)				Diabetic (140)	Non-diabetic (60)	
GAS6 (cut-off: <174.65 pg/mL)	Normal kidney	117 (90%)	0 (0%)	0.863	GAS6 (cut-off: <213 pg/mL)	Diabetic	106 (75.7%)	7 (11.7%)	0.568
	Nephropathy	13 (10%)	70 (100%)			Non-diabetic	34 (24.3%)	53 (88.3%)	
		Fisher’s Exact, *p* < 0.0001					χ^2^ = 70.10, *p* < 0.0001		
AXL (cut-off: <149.65 pg/mL)	Normal kidney	119 (91.5%)	9 (12.9%)	0.782	AXL (cut-off: <231.6 pg/mL)	Diabetic	112 (80%)	12 (20%)	0.558
	Nephropathy	11 (8.5%)	61 (87.1%)			Non-diabetic	28 (20%)	48 (80%)	
		χ^2^ = 122.257, *p* < 0.0001					χ^2^ = 64.177, *p* < 0.0001		
Combined GAS6/AXL (both cut-offs)	Normal kidney	129 (99.2%)	9 (12.9%)	0.887	Combined GAS6/AXL (both cut-offs)	Diabetic	91 (65%)	2 (3.3%)	0.504
	Nephropathy	1 (0.8%)	61 (87.1%)			Non-diabetic	49 (35%)	58 (96.7%)	
		Fisher’s Exact, *p* < 0.0001					Fisher’s Exact, *p* < 0.0001		
GAS6-AS1 (cut-off: >1.203 folds)	Normal kidney	69 (53.1%)	26 (37.1%)	0.143	GAS6-AS1 (cut-off: >1.203 folds)	Diabetic	83 (59.3%)	22 (36.7%)	0.194
	Nephropathy	61 (46.9%)	44 (62.9%)			Non-diabetic	57 (40.7%)	38 (63.3%)	
		χ^2^ = 4.632, *p* = 0.031					χ^2^ = 8.617, *p* = 0.0003		
GAS6-DT (cut-off: >11.21 folds)	Normal kidney	130 (100%)	20 (28.6%)	0.765	GAS6-DT (cut-off: >3.29 folds)	Diabetic	138 (98.6%)	2 (3.3%)	0.952
	Nephropathy	0 (0%)	50 (71.4%)			Non-diabetic	2 (1.4%)	58 (96.7%)	
		Fisher’s Exact, *p* < 0.0001					Fisher’s Exact, *p* < 0.0001		

Data are expressed as *n* (%), analyzed by chi-square or Fisher’s Exact test and Cohen’s kappa.

Significance was considered at *p* < 0.05 (two-tailed) χ^2^.

### In silico interactions with biomolecules related to DM or DN

**Fig. 3:**
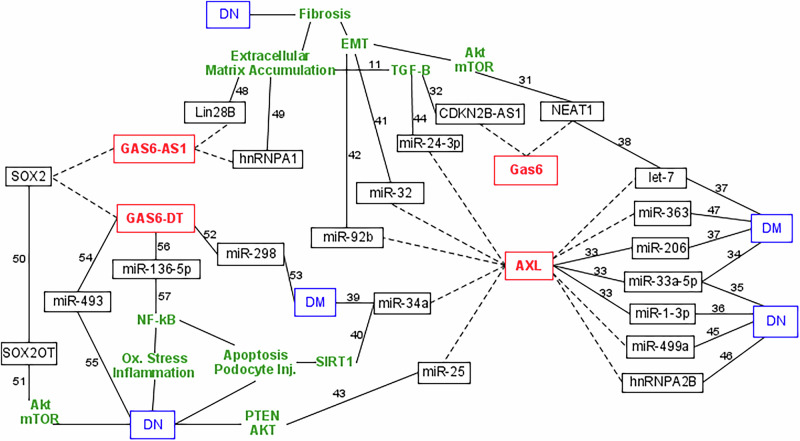
The possible interactions between GAS6, AXL, GAS6-AS1, GAS6-DT, and other biomolecules that may be related to diabetes mellitus and diabetic nephropathy. This figure was drawn using PathVisio biological pathway editor, version 3.0.0+. Databases used for predictions were NPInter, miRTarBase, and TargetScan. Dashed lines represent predicted interactions. Solid lines represent verified interactions with references clarified as numbers (numbering 11 to 57 is the same as in the main manuscript). PubMed identifiers of references (PMID) (more details can be found under references): [11], 38390204; [31], 30515796; [32], 34649592; [33], 29572052; [34], 21576456; [35], 27613243; [36], 38935970; [37], 30584410; [38], 31570982; [39], 37113991; [40], 38270305; [41], 32959695; [42], 32215042; [43], 28923913; [44], 37251672; [45], 34934434; [46], 36765416; [47], 32104036; [48], 31485601; [49], 35121168; [50], 29321583; [51], 34238205; [52], 30394665; [53], 32664305; [54], 31933797; [55], 36722214; [56], 35793478; [57], 10.1166/mex.2023.2424. DM diabetes mellitus, DN diabetic nephropathy, EMT epithelial–mesenchymal transition.

Investigating the possible interactions between our parameters and other biomolecules that may be related to the diseases under study, several predicted interactions have been found through different database searching and were proven by literature to be related to DM or DN (Fig. 3 and Supplementary Table 3).

Pathways associated with GAS6/AXL that may be implicated in DN were illustrated in Supplementary Fig. 2, as obtained from the KEGG PATHWAY Database.

Applying the different bioinformatics tools of nsSNP analysis to AXL, GAS6-AS1, and GAS6-DT genes showed no deleterious nsSNPs, while GAS6 gene had only GAS6 rs1803628 as a potential deleterious SNP. In GAS6 rs1803628, the alteration of the nucleotide in position 13:114530114 from cytosine to guanine (C>G) changed the amino acid at position 444 of GAS6 protein from cysteine to tryptophan (C 444 W). This affected the protein 3D structure and caused destabilization of the protein due to the bigger size of the mutant residue and loss of the cysteine bridge. In addition, the mutation is located within the Laminin G-like 1 domain, abolishing its function. The SNP is also located near a highly conserved position, where mutations are usually damaging for the protein.

## Discussion

DN is a scientific research priority around the world because of its high prevalence and poor prognosis, even in freshly diagnosed patients with diabetes. Almost one-third of patients with type 2 diabetes accompanied by both normoalbuminuria and microalbuminuria showed a rapid decline in kidney function. Therefore, it is important to find better clinicopathological predictors of DN in patients with diabetes than proteinuria [19]. Previous studies suggesting a role of GAS6/AXL axis in DN came with varying results. Accordingly, in this study we aimed at verifying whether GAS6, AXL, Gas-AS1, and GAS6-DT can be used as early markers for DN. The enrolled 140 patients had type 2 diabetes, 70 had normal kidney functions (DM group), while 70 started showing kidney dysfunction represented in this study as microalbuminuria (DN group). They were compared to 60 apparently healthy sex- and age-matched controls.

In the current investigation, plasma GAS6 and AXL levels were notably downregulated in patients with diabetes than in controls, in accordance with several previous studies in Taiwan and Turkey [20, 21], and they were significantly lower in patients with diabetes and microalbuminuria than in those with normoalbuminuria. The gradually decreasing level with diabetes development and DN progression was separately described in Chinese population for GAS6 [8] and AXL [22]. This very same pattern was reported in a Chinese study on another microvascular complication of diabetes, namely, carotid atherosclerosis [23]. Other studies on DN reported upregulation of GAS6 in a Nigerian population [9], as well as upregulated AXL level in a study in Norway [24]. These discrepancies may be attributed to different genetic dispositions in different populations, or different study design and sample size included.

The significant moderate negative correlations that we found between GAS6 and AXL, on one hand, and the continuous variables (HbA1c, fasting glucose, creatinine, and albuminuria), on the other hand, also confirm their association with DN. Similar correlations were previously observed with fasting glucose [20, 23] and with kidney functions presented as serum albumin, glomerular filtration rate, and albuminuria [21, 22].

The results reported herein imply a protective effect of GAS6 and AXL in DN. Their protective effect was previously suggested in other kidney disorders. In a study on sepsis-induced acute kidney injury, GAS6 exerted protective effects through reducing renal tissue apoptosis, and suppressing inflammation and oxidative stress by inhibiting the downstream mediators such as nuclear factor-kappa B (NF-κB) [25], taking into consideration that targeting of the NF-κB pathway had renoprotective effects [26]. In another animal model with renal ischemia/reperfusion injury, treatment with recombinant GAS6 provided renoprotective activity, through reducing mRNA levels of the inflammation-triggered enzymes, cyclooxygenase-2 and inducible nitric oxide synthase [27]. Data obtained from the KEGG PATHWAY Database suggest the implication of GAS6/AXL in several pathways related to DN, including PI3K/AKT/mTOR, Jak/STAT, MAPK, apoptosis, and epithelial–mesenchymal transition.

Evaluating the diagnostic power of the parameters under investigation revealed an excellent ability of GAS6 and AXL to differentiate patients with diabetes accompanied by nephropathy from patients with diabetes and normal kidney and from controls, with excellent sensitivity and specificity. Combining GAS6 and AXL even augmented their diagnostic power than either alone. GAS6 showed excellent agreement with the conventional diagnostic parameters, plasma creatinine and microalbuminuria, while AXL demonstrated substantial agreement. A previous study reported the usefulness of GAS6 [8] as a diagnostic of DN but with different cut-offs. However, AXL diagnostic power for DN was observed only in combination with other TAM receptors but not alone [22].

In the present study, lncRNA GAS6-DT showed remarkably higher levels in the DN group than in the DM group, which, in turn, displayed significantly higher GAS6-DT levels than the controls. Fair-to-moderate significant correlations between GAS6-DT and the continuous parameters (HbA1c, fasting glucose, creatinine, and albuminuria) were illustrated. Evaluating its diagnostic power, GAS6-DT could discriminate patients with diabetes from controls with excellent sensitivity and specificity. It also showed good to excellent discrimination of DN from DM (patients with diabetes and normal kidney) and from controls, with high sensitivity and specificity. As a diagnostic test for DN, GAS6-DT had substantial agreement with microalbuminuria and creatinine. As a diagnostic test for DM, GAS6-DT had excellent agreement with HbA1c and fasting glucose. In our study, GAS6-DT was inversely correlated to GAS6 and AXL, which is contradictory to some other studies reporting that GAS6-DT was a positive regulator of GAS6 and its TAM receptors [15]. Considering the limited studies on GAS6-DT, and that diverse regulatory effects on gene expression at variable levels may arise from antisense RNA interactions with its target gene [28], more studies are required to elucidate the precise mechanism underlying the effect of GAS6-DT on GAS6 expression.

GAS6-AS1 level showed an inverse correlation with GAS6 and AXL levels, which is consistent with previous reports [13, 14], suggesting that GAS6-AS1 could control its cognate sense gene GAS6 expression, at the transcriptional or translational levels [29]. We also found that GAS6-AS1 showed significantly higher values in the DN group in comparison with controls, but the increase was not significant compared to the DM group. GAS6-AS1 showed only weak correlations to other parameters (HbA1c, fasting glucose, creatinine, and albuminuria). GAS6-AS1 had a statistically significant but poor discriminative power to differentiate patients with nephropathy from controls, but it could not discriminate normal kidney DM from controls nor from DN. Its agreement with the conventional diagnostic parameters of DM and DN was very weak. Previous studies on GAS6-AS1 in DM or DN are rare; it was only suggested to mechanically repress glucose transporter GLUT1 expression, which is an important regulator of glucose metabolism [30].

Harnessing lncRNA interactions may suggest pioneering ncRNA-based therapeutics for kidney diseases among a multitude of medical conditions [2]. The predicted in silico interactions of the currently studied parameters with other biomolecules provided an insight into possible mechanisms that may link them to diabetes and DN (as clarified in Fig. 3). GAS6 was predicted to interact with two lncRNAs that were clinically proven to affect DN fibrosis. The first is the nuclear enriched abundant transcript 1 (NEAT1) that regulated Akt/mTOR signaling, which is a pathway associated with EMT in renal fibrosis progression [31]. The second is the cyclin-dependent kinase inhibitor 2B antisense RNA 1 (CDKN2B-AS1) that promoted fibrosis via transforming growth factor-β (TGF-β) signaling [32], which, in turn, increased the synthesis and deposition of extracellular matrix, eventually causing glomerulosclerosis and tubulointerstitial fibrosis in DN [11].

AXL had many important predicted interactions that may explain its association with diabetes and DN. Some predicted miRNAs were already proven to have binding sites on AXL: miR-33a-5p, miR-1-3p, and miR-206 [33]. miR-33a played a role in insulin signaling [34] and in DN progression [35]. mir-1-3p was involved in diabetic complications with significantly altered levels in renal tissue [36]. hsa-mir-206 was associated with type 2 diabetes progression [37].

Other predicted interactions with AXL have not yet been clinically confirmed, so more laboratory studies are still needed to validate these interactions. For example, AXL had predicted interactions with certain members of the let-7 family, which were related to T2DM [37] and were regulated by NEAT1 [38], already associated with DN fibrosis [31]. Another is miR-34a, which has been related to diabetes [39], and to apoptosis and podocyte injury in DN through targeting SIRT1 [40]. Also, miR-32 [41] and miR-92b [42] induced the progression of EMT and fibrosis in DN. Another is the protective miR-25, which was significantly decreased in DN patients, likely acting through PTEN/AKT pathway activation [43]. Similarly, the reno-protective miR-24-3p, that downregulated fibrotic/inflammatory genes (Collagen IV and TGF-β), was lower in DN [44]. AXL also interacted with several other miRNAs related to DN: miR-499a [45] and heterogeneous nuclear ribonucleoprotein A2B1 (hnRNPA2B1) [46], as well as its predicted interaction with miR-363 that was related to insulin resistance [47].

GAS6-AS1 was predicted to interact with LIN28B [48] and hnRNPA1 [49], which boosted deposition of extracellular matrix and development of DN fibrosis. Both GAS6-AS1 and GAS6-DT were predicted to interact with SOX2, which is repressed by lncRNA SOX2-overlapping transcript (SOX2OT) [50], which may affect the pathogenesis of DN [51].

GAS6-DT has been clinically confirmed to interact with some miRNAs that were related to DM and DN. GAS6-DT directly sponged miR-298 [52], which was a suggested diagnostic for T2DM prediction in patients with prediabetes [53]. GAS6-DT could regulate miR- 493 [54], which was related to DN [55]. The interactions between GAS6-DT and miR-136-5p were validated [56]. miR-136-5p regulated the NF-κB pathway, oxidative stress, fibrosis, and apoptosis in DN [57].

Therapeutically targeting the GAS6/AXL pathway is still a promising avenue for future research. Rare deleterious mutations in GAS6 and AXL proteins, as proven by our SNP analysis and a recent study [58], makes them attractive therapeutic targets with low risk of developing drug resistance. Modulating the pathway in DN has been investigated in animal models [27, 59]. Human clinical trials are focusing on AXL inhibitors mainly in cancer [58, 60]. Thus, pathway stimulation still needs to be investigated, as well as studying the efficacy in other diseases such as DN. RNA-based therapies for certain kidney diseases are already in the preclinical and clinical trials [2]. However, there is no direct published evidence on treatments targeting GAS6-AS1 or GAS6-DT as interventions for DN or other diseases, which makes it an interesting path for future research.

Several study limitations should be acknowledged. In our work, the relatively small sample size, the inclusion of only Egyptians, and studying both sexes together are among the study constraints. Not validating the in silico results in the laboratory is another limitation. Our study focused specifically on DN; while this does not rule out the involvement of the studied markers in other diabetic complications, our findings highlight their potential relevance within the renal context.

## Conclusions

**Fig. 4:**
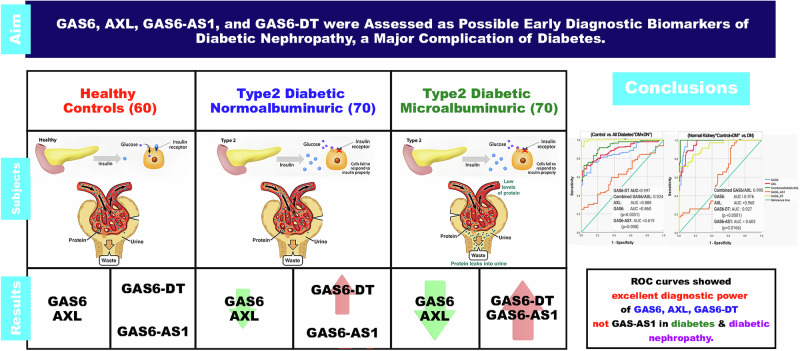
Illustrated summary of study findings.

The results of the current study highlight the role of GAS6/AXL pathway in the course of DM and DN, where GAS6, AXL, and GAS6-DT significantly changed values from controls to DM, and significantly changed further in DN, exhibiting remarkable diagnostic power for both diseases, as illustrated in Fig. 4. To date, this is the first study to report the diagnostic power of AXL and GAS6-DT in DN and DM.

Although the sample size had sufficient power, further rigorously designed trials are recommended to replicate our findings in larger datasets and in diverse populations. Future investigations are warranted to explore the therapeutic potential of targeting this pathway in the management of DN, to conduct mechanistic studies validating the role of GAS6-DT in DN, and to confirm the predicted interactions with other biomolecules as suggested underlying mechanisms. Also, further studies comparing GAS6/AXL expression across different diabetic complications would be valuable in determining their specificity to DN. Additionally, the next level of studies required to follow on is biomarker validation studies, to develop a pre-specified protocol detailing specimen collection, handling, storage, and clinical endpoints; as well as to establish technical performance characteristics: sensitivity, specificity, accuracy, precision, and reproducibility; where appropriate study designs are essential for analytical and clinical validation, to ensure that biomarkers are reliable, reproducible, and clinically meaningful before implementation in practice.

## Supplementary information

Supplementary data

## Data Availability

The data that support the findings of this study are available in the “Materials and methods,” “Results,” and Supplementary Materials of this article. The raw data analyzed during the study are available at: https://1drv.ms/x/s!AmMXCWTKwZD5gZVgF3e6Szj6yOMTRw?e=YT0VIh.
