# Supplementary material for: Association of GAS6, AXL, and GAS6-AS lncRNAs with nephropathy in Egyptian patients with type 2 diabetes mellitus: a case–control observational study
Source: Nutr Diabetes. 2025 Nov 13;15:45. doi: 10.1038/s41387-025-00400-y (PMC12615580; doi:10.1038/s41387-025-00400-y)
Supplement: Supplementary file 1 — Supplementary data [file 41387_2025_400_MOESM1_ESM.docx]

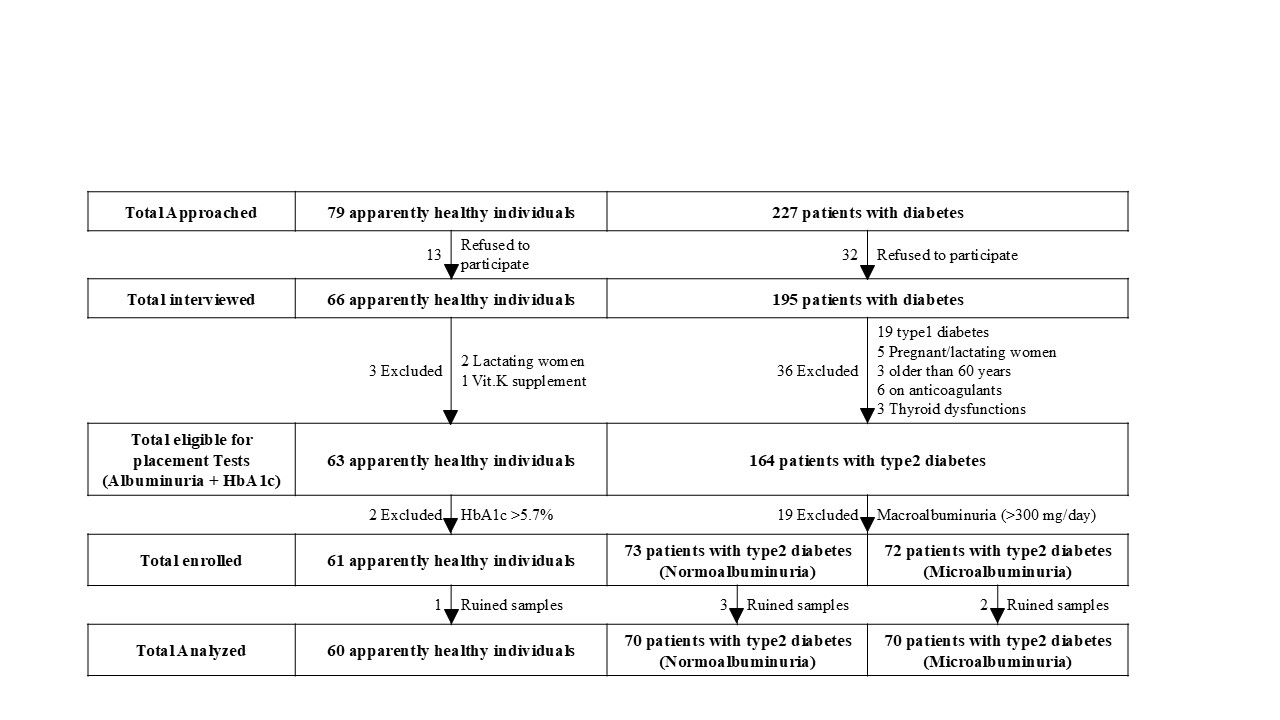


Supplementary Figure 1. Participants’ flow chart


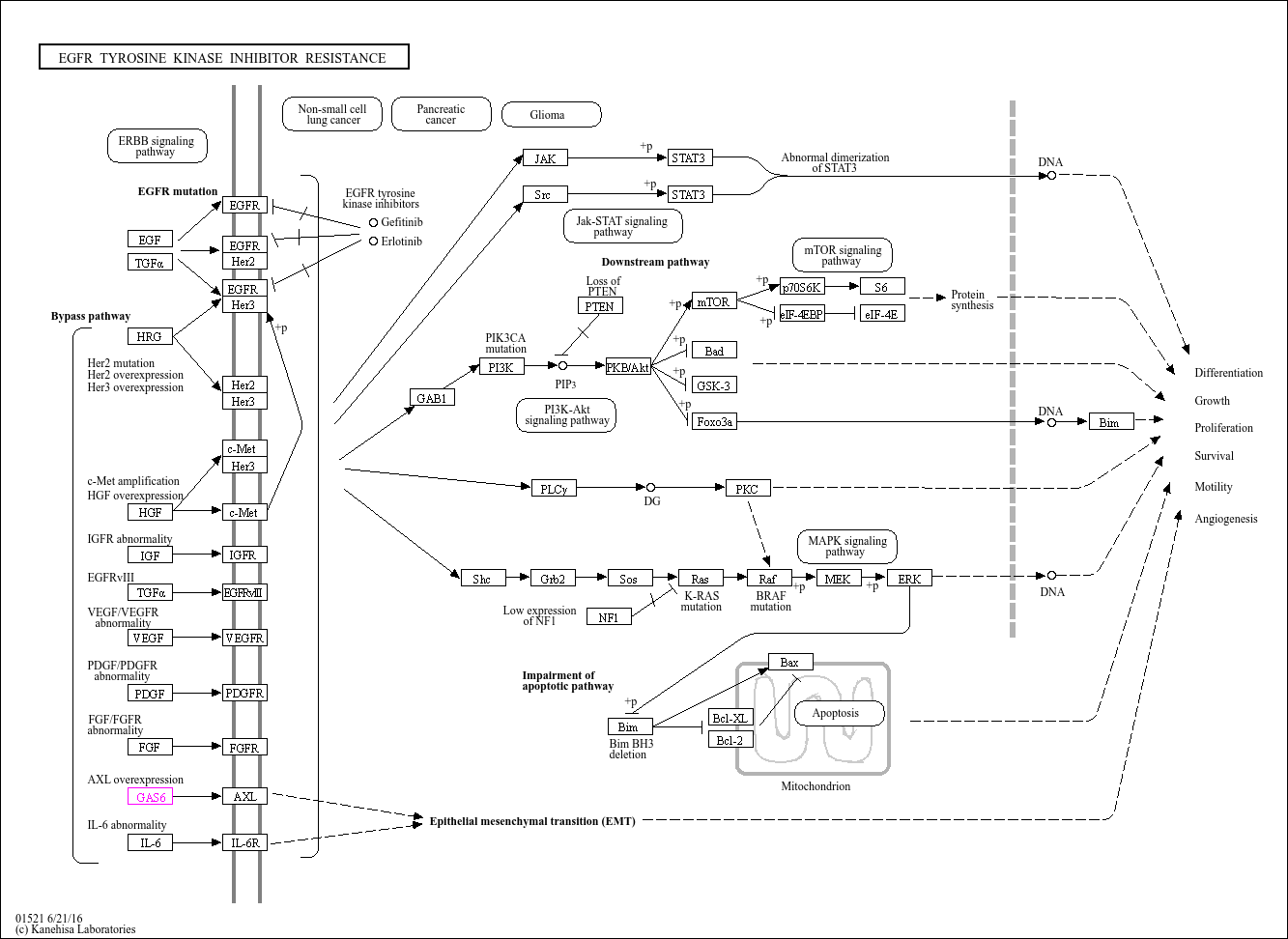


Supplementary Figure 2. Pathways associated with GAS6/AXL that maybe implicated in diabetic nephropathy, as obtained from KEGG PATHWAY database

Supplementary Table 1. Spearman correlations of continuous parameters

|  | | HbA1c | F_Glucose | Creatinine | Albuminuria | AXL | GAS6-AS1 | GAS6-DT |
| --- | --- | --- | --- | --- | --- | --- | --- | --- |
| GAS6 | rho | -0.755 | -0.639 | -0.641 | -0.676 | 0.588 | -0.220 | -0.526 |
|  | p | <0.0001 | <0.0001 | <0.0001 | <0.0001 | <0.0001 | 0.002 | <0.0001 |
| AXL | rho | -0.724 | -0.659 | -0.632 | -0.648 |  | -0.145 | -0.506 |
|  | p | <0.0001 | <0.0001 | <0.0001 | <0.0001 |  | 0.042 | <0.0001 |
| GAS6-AS1 | rho | 0.254 | 0.192 | 0.298 | 0.235 |  |  | -0.020 |
|  | p | <0.0001 | <0.0001 | <0.0001 | 0.001 |  |  | 0.779 |
| GAS6-DT | rho | 0.608 | 0.571 | 0.494 | 0.559 |  |  |  |
|  | p | <0.0001 | <0.0001 | <0.0001 | <0.0001 |  |  |  |
| Correlations are expressed as rho: Spearman coefficient. F: fasting, HbA1c: glycosylated hemoglobin.  Significance was considered at p<0.05 (two-tailed). | | | | | | | | |

***Supplementary Table 2. Receiver operating characteristic (ROC) curve details of parameters related to the GAS6/AXL pathway***

|  | **Differentiated groups** | **AUC** | **p** | **95% Confidence Interval** | | **Cut-off** | | |  |  |
| --- | --- | --- | --- | --- | --- | --- | --- | --- | --- | --- |
|  |  |  |  | **Lower Bound** | **Upper Bound** | **Cut-off** | **Sensitivity** | **Specificity** |  |  |
| **GAS6** (pg/mL) | Control (60) DM (70) | 0.722 | <0.0001 | 0.635 | 0.809 | 213 | 51.43% | 88.33% |  |  |
|  | Control (60) All diabetics (140) | 0.860 | <0.0001 | 0.811 | 0.910 | 213 | 75.71% | 88.33% |  |  |
|  | Control (60) DN (70) | 0.998 | <0.0001 | 0.995 | 1.000 | 174.65 | 100.00% | 98.33% |  |  |
|  | Normal kidney (130) Nephropathy (70) | 0.976 | <0.0001 | 0.959 | 0.993 | 174.65 | 100.00% | 90.00% |  |  |
|  | DM (70) DN (70) | 0.957 | <0.0001 | 0.926 | 0.988 | 174.65 | 100.00% | 82.86% |  |  |
| **AXL** (pg/mL) | Control (60) DM (70) | 0.779 | <0.0001 | 0.701 | 0.857 | 231.6 | 60.00% | 80.00% |  |  |
|  | Control (60) All diabetics (140) | 0.888 | <0.0001 | 0.845 | 0.932 | 231.6 | 80.00% | 80.00% |  |  |
|  | Control (60) DN (70) | 0.997 | <0.0001 | 0.993 | 1.000 | 184.25 | 100.00% | 96.67% |  |  |
|  | Normal kidney (130) Nephropathy (70) | 0.960 | <0.0001 | 0.938 | 0.982 | 149.65 | 87.14% | 91.54% |  |  |
|  | DM (70) DN (70) | 0.928 | <0.0001 | 0.890 | 0.967 | 149.65 | 87.14% | 84.29% |  |  |
| Combined GAS6-Ax1 | Control (60) DM (70) | 0.849 | <0.0001 | 0.781 | 0.916 |  | 82.86% | 78.33% |  |  |
|  | Control (60) All diabetics (140) | 0.924 | <0.0001 | 0.888 | 0.960 |  | 89.29% | 80.00% |  |  |
|  | Control (60) DN (70) | 1.000 | <0.0001 | 1.000 | 1.000 |  | 100.00% | 100.00% |  |  |
|  | Normal kidney (130) Nephropathy (70) | 0.995 | <0.0001 | 0.989 | 1.000 |  | 100.00% | 98.46% |  |  |
|  | DM (70) DN (70) | 0.992 | <0.0001 | 0.980 | 1.000 |  | 100.00% | 97.14% |  |  |
| **GAS6-AS1** (fold change) | Control (60) DM (70) | 0.578 | 0.127 | 0.477 | 0.679 | Non-significant | | |  |  |
|  | Control (60) All diabetics (140) | 0.619 | 0.008 | 0.540 | 0.697 | 1.203 | 59.29% | 63.33% |  |  |
|  | Control (60) DN (70) | 0.659 | 0.002 | 0.565 | 0.748 | 1.203 | 62.86% | 63.33% |  |  |
|  | Normal kidney (130) Nephropathy (70) | 0.603 | 0.0166 | 0.523 | 0.683 | 1.203 | 62.86% | 53.08% |  |  |
|  | DM (70) DN (70) | 0.554 | 0.266 | 0.454 | 0.655 | Non-significant | | |  |  |
| **GAS6-DT** (fold change) | Control (60) DM (70) | 0.995 | <0.0001 | 0.998 | 1.000 | 3.29 | 97.14% | 96.67% |  |  |
|  | Control (60) All diabetics (140) | 0.997 | <0.0001 | 0.993 | 1.000 | 3.29 | 98.57% | 96.67% |  |  |
|  | Control (60) DN (70) | 0.999 | <0.0001 | 0.997 | 1.000 | 3.29 | 100.00% | 96.67% |  |  |
|  | Normal kidney (130) Nephropathy (70) | 0.927 | <0.0001 | 0.890 | 0.963 | 11.21 | 71.43% | 100.00% |  |  |
|  | DM (70) DN (70) | 0.864 | <0.0001 | 0.802 | 0.927 | 11.21 | 71.43% | 100.00% |  |  |

Supplementary Table 3. Examples of interactions with the biomolecules under study, which were proven by literature to be related to DM or DN

| Parameter | | Predicted Interaction ncRNA | | Database | | Literature  Related to DN | Literature  Related to DM | |
| --- | --- | --- | --- | --- | --- | --- | --- | --- |
| GAS6 | | NEAT1 | | NPInter | | (31) |  | |
| GAS6 | | CDKN2BAS | | NPInter | | (32) |  | |
| AXL | | miR-33a  a confirmed interaction(33) | | TargetScan | | (35) | (34) | |
| AXL | | miR-1  a confirmed interaction(33) | | TargetScan | | (29) |  | |
| AXL | | miR-206  a confirmed interaction(33) | | TargetScan | |  | (37) | |
| AXL | | let-7f-2-3p | | NPInter | | (38) | (37) | |
| AXL | miR-34a | | miRTarBase  TargetScan | | (40) | | | (39) |
| AXL | miR-32 | | TargetScan | | (41) | | |  |
| AXL | miR-92b | | TargetScan | | (42) | | |  |
| AXL | miR-25 | | TargetScan | | (43) | | |  |
| AXL | miR-24 | | TargetScan | | (44) | | |  |
| AXL | miR-449a | | TargetScan | | (45) | | |  |
| AXL | hnRNPA2B1 | | NPInter | | (46) | | |  |
| AXL | miR-363 | | TargetScan | |  | | | (47) |
| GAS6-AS1 | hnRNPA1 | | NPInter | | (49) | | |  |
| GAS6-AS1 | LIN28B | | NPInter | | (48) | | |  |
| GAS6-AS1 GAS6-DT | SOX2 | | NPInter | | (50,51) | | |  |
| GAS6-DT | miR‐298  a confirmed interaction(52) | |  | |  | | | (53) |
| GAS6-DT | miR- 493  a confirmed interaction(54) | |  | | (55) | | |  |
| GAS6-DT | miR-136-5p  a confirmed interaction(56) | |  | | (57) | | |  |
| Nuclear Enriched Abundant Transcript 1 (NEAT1)  cyclin-dependent kinase inhibitor 2B antisense RNA 1 (CDKN2B-AS1)  heterogeneous nuclear ribonucleoprotein (hnRNP) | | | | | | | | |
